# Bridging the divide between medical school and clinical practice: identification of six key learning outcomes for an undergraduate preparatory course in radiology

**DOI:** 10.1186/s13244-021-00971-1

**Published:** 2021-02-12

**Authors:** Thabisile Simelane, David J. Ryan, Slavi Stoyanov, Deirdre Bennett, Mark McEntee, Michael M. Maher, Colm M. P. O’Tuathaigh, Owen J. O’Connor

**Affiliations:** 1 Department of Radiology, University Hospital Kerry, Kerry, Ireland; 2 Department of Radiology, Cork University Hospital, Wilton, Cork Ireland; 3 Open University of the Netherlands, 177, Valkenburgerweg, 6401 DL Heerlen, The Netherlands; 4 Medical Education Unit, School of Medicine, University College Cork, Cork, Ireland; 5 Department of Radiography, School of Medicine, University College Cork, Cork, Ireland; 6 Department of Radiology, School of Medicine, University College Cork, Cork, Ireland

**Keywords:** Preparedness for clinical practice, Radiology, Internship, Radiologists, Group concept mapping, Medical school, Interventional radiology

## Abstract

**Background:**

There exists a significant divide between what is learnt in medical school and subsequently what is required to practice medicine effectively. Despite multiple strategies to remedy this discordance, the problem persists. Here, we describe the identification of a comprehensive set of learning outcomes for a preparation for practice course in radiology.

**Methods:**

Assessment of interns’ readiness to interact with the radiology department was conducted using a national survey of both interns and radiologists. In parallel, group concept mapping (GCM) which involves a combination of qualitative and quantitative techniques was used to identify the shared understanding of participants from a diverse range of medical specialties regarding what topics should be included in an intern preparatory course for interacting with the radiology department.

**Results:**

The survey demonstrated that most interns and radiologists felt that undergraduate medical training did not prepare interns to interact with the radiology department. GCM identified six learning outcomes that should be targeted when designing a preparatory module: requesting investigations; clinical decision support; radiology department IT and communication; adverse reactions and risks; interpretation of radiology results and urgent imaging. The thematic clusters from the group concept mapping corroborated the deficiencies identified in the national survey.

**Conclusion:**

We have identified six key learning outcomes that should be included in a preparation for practice module in radiology. Future courses targeting these thematic clusters may facilitate a smoother transition from theory to practice for newly graduated doctors.

## Key points

- National survey revealed that undergraduate medical training does not prepare interns to interact with the radiology department.
- Six key learning outcomes that should be targeted when designing a preparatory module were identified.
- Thematic clusters from the group concept mapping corroborated the deficiencies identified in the national survey.

## Introduction

As doctors, we have a duty of care to our patients with continual reflection on one’s practice being essential to identify areas in need of improvement. One such area that results in preventable increased risk for patients is the annual trainee change over, labelled the *July effect* when newly graduated doctors begin their clinical careers [1, 2]. Although multifactorial, a recurring theme that has emerged to explain this phenomenon is the knowledge discordance that exists between what is learnt in medical school and subsequently what is required to practice medicine effectively. It is acknowledged that preparation-for-practice and work orientation modules should be designed to prepare junior doctors for this transition, thereby increasing patient safety and quality of care [2]. Unfortunately, despite these efforts, the problem persists [3–7]. This may be explained in part by the focus of preparation courses on a singular domain such as clinical procedural skills which do not address the problem holistically.

The scientific discipline of chemistry has been described as the central science; in modern healthcare, radiology is central to patient management. A wide range of specialties depend on input from imaging data to facilitate optimal patient management [8]. Recognising this, multiple studies have highlighted the need for inclusion of radiology in undergraduate training [9–11]. It is suggested that a strong undergraduate training in radiology will result in efficient, and improved patient care, thus reducing unnecessary imaging examinations, minimising the potential harm to patients and reducing costs [10]. However, consistent with what is observed with other disciplines, the undergraduate training received in radiology does meet the learning needs required for clinical practice [9, 12]. In addition, preparation-for-practice courses have placed little emphasis on radiology [3–7]. Given the central role that medical imaging commands in patient care, engagement with diagnostic imaging and interventional radiology is a major part of daily life for newly qualified doctors which presents both challenges and opportunities for error.

Therefore, we sought to identify areas that should be targeted when designing a radiology preparatory module to facilitate a smooth transition from medical school to clinical practice.

## Methods

The study was split into two components; assessment of interns’ readiness to interact with the radiology department using a quantitative national survey and group concept mapping to evaluate what topics should be covered in a preparatory course. Survey design was performed without knowing the results of the GCM.

### Part 1: survey of interns’ readiness to interact with the radiology department

#### Questionnaire design

A novel questionnaire was designed to cover the following domains: (a) Demographic characteristics for intern and radiologist participants; (b) Perceived adequacy of undergraduate radiology teaching and how this prepares interns for clinical practice; (c) Radiology and working as an intern, based on the competencies as outlined in the radiology undergraduate curriculum. Competencies assessed were understanding various imaging modalities, their appropriateness, indications, and limitations; radiation protection; use of contrast media; communication in radiology; checking and acting on radiology results, and handover. All items were formatted using a six-point Likert scale ranging from ‘strongly agree’ to ‘strongly disagree’. This scale was selected based on its use in the Preparation for Practice Questionnaire (PHPQ), an instrument that has been previously used by medical schools to assess their graduates’ clinical capabilities [13, 14].

The wording of items administered to both interns and radiologists were matched as far as possible to facilitate comparisons. Validity of the questionnaire was checked prior to distribution by eight consultant radiologists who assessed whether the questions were appropriate, clearly designed, and suitable for answering the research question. An open comments section was also included where participants were asked to highlight any topics not covered in the survey which they considered important for inclusion in an intern preparatory course.

#### Study population

The quantitative survey study population was (a) all interns in the national intern training networks and (b) all nationally registered consultant radiologists, and radiology registrars in 2017. Ethical approval was obtained from the relevant ethics committees affiliated with each of the intern training networks. Questionnaires were also distributed by the national regulatory body for radiologists which keeps a register of all practicing radiologists and works closely with the Medical Council to maintain high national professional standards. As the surveys were distributed to interns across all the training networks, all geographical regions were therefore represented in the initial invitation to participate. We did not ask participants to identify their training network as this was deemed to increase the potential for identification of participants when added to the other demographic details collected in the survey. For the radiologist survey, we did not collect data about respondents’ clinical site to avoid the potential for self-identification.

#### Questionnaire data analysis

Summary statistics were used to analyse Likert-scale responses and ratio scale measurements for all respondents. Frequency analysis was used to describe and summarise questionnaire items, which required a categorical response. Kruskal–Wallis analysis of variance (ANOVA) and/or Mann–Whitney *U* tests were employed to carry out univariate comparisons where the outcome variable(s) consisted of Likert-scale question responses. Statistical analyses were carried out using IBM SPSS 20 (IBM, New York, NY, USA).

### Part 2: group concept mapping (GCM)

GCM is an integrated mixed-method research approach which involves a combination of qualitative and quantitative techniques in order to identify an expert group’s understanding about a topic [15]. Given the complexity of the two-way interaction between radiology and every specialty within the hospital, group concept mapping (GCM) was used to incorporate the opinion of clinicians from multiple specialties [13, 14]. Participants were asked to generate ideas in response to the focus prompt: ‘If I was asked to design an intern preparatory course for interacting with the radiology department, the following topics would be covered’. The primary outcome measure in this analysis was the list of topics derived from the GCM which respondents would include when asked to design an intern preparatory course for interacting with the radiology department. The secondary outcome measures were the strategy ratings in terms of importance and ease of inclusion in a preparation for practice module. See Additional file 1 for additional details on the GCM methodology and data analysis.

## Results

### National quantitative survey (Supporting data in Additional file 1: Appendix A)

#### Response rate and population demographics

Seven hundred and thirty-three interns were invited to participate in this study, and a total of 100 responses were received. This corresponds to a response rate of 14%. Fifty-two percent of intern respondents were female. For radiologists, out of a total of 350 contacted, 50 responded, denoting a response rate of 14%. Seventy-two percent (*n* = 36) of respondent were male. Ninety-two percent (*n* = 46) worked in teaching hospitals with 74% (*n* = 37) being consultant radiologists. The remainder were specialist trainees in radiology.

#### Undergraduate radiology teaching and preparedness for clinical practice

Most interns, 67%, felt that they received adequate radiology instruction during their undergraduate training with 55% reporting ‘adequate’ knowledge of radiology when compared to other subjects (Additional file 1: Table S1 and Additional file 1: S2). Concordantly, most radiologists, 64%, felt that interns received adequate radiology teaching during their undergraduate training (Additional file 1: Table S3). Forty-three percent of interns received formal radiation protection teaching, 35% were informally taught, and 22% had little or no exposure to radiation protection. Sixty-six percent of interns indicated a clear understanding of the different imaging modalities (Additional file 1: Table S4).

However, most intern respondents, 66%, felt that undergraduate medical training did not prepare them for interacting with the radiology department (Additional file 1: Table S5). No significant difference was observed between interns and radiologists with respect to how they rated intern preparedness to interact with the radiology department (*p* > 0.05) (Additional file 1: Table S6).

#### Radiology and working as an intern—core competencies assessment

##### Requesting imaging modalities

Despite most interns indicating that they understood the different imaging modalities as an undergraduate; as an intern, 53% reported frequent uncertainty regarding the indication for radiology examinations when completing an investigation request (Additional file 1: Table S7). Most radiologists, 92%, concurred with this deficiency (Additional file 1: Table S8). In addition, radiologists were significantly less likely than interns themselves to express confidence in interns’ awareness of the indications across all imaging modalities: plain film (*U* = 1345, *z* = − 4.10, *p* < 0.001), ultrasound (*U* = 1392, *z* = − 3.42, *p* = 0.001), CT (*U* = 1412.5, z = − 3.20, *p* = 0.001), and MRI (*U* = 1164.5, *z* = − 3.07, *p* = 0.002) (Additional file 1: Table S9 and Additional file 1: S10).

##### Radiation protection and contrast media in radiology

The majority, 77%, of interns were not familiar with the 10-day rule when imaging patients of childbearing age (Additional file 1: Table S11). Concordantly, most radiologists, 78%, felt that interns had inadequate understanding of radiation protection as it applies in radiological imaging (Additional file 1: Table S12). Regarding contrast media, 53% of interns felt that they had adequate understanding of the use of contrast medium in radiology investigations (Additional file 1: Table S13). By comparison, 86% radiologists reported that they felt that interns did not understand the appropriate use of contrast media for radiology investigations with this difference being statistically significant (*U* = 339, z = − 6.46, *p* < 0.001) (Additional file 1: Table S14).

##### Communication in radiology

Most interns perceived ‘getting the study done’, and ‘communication with the radiologist or radiographer’ as the most difficult challenge they face when dealing with the radiology department (Additional file 1: Table S15). Just over half, 52%, intern respondents indicated that the radiology department was approachable (Additional file 1: Table S16). In contrast, radiologists were significantly more likely to rate their department as approachable relative to interns (*U* = 1318, *z* = 4.82, *p* < 0.001) (Additional file 1: Table S17). Consistent with the intern perception, only 3% of interns reported that they would ask a radiologist if they needed guidance regarding the choice of imaging modality. Most interns, 74%, would ask a colleague, 11% would look it up on the internet, and 5% would ask their consultant (Additional file 1: Table S18). The internet sources for guidance on the choice of imaging modality used by respondents were search engines such as google (46%), specific radiology sites (32%) and 22% reported using other sources (Additional file 1: Table S19); 11% had difficulty using the online request system and 26% encountered problems when preparing patients for a test or intervention (Additional file 1: Table S15).

##### Checking and acting on radiology results and handover

When asked about checking radiology results, 87% reported that they frequently check the radiology results (Additional file 1: Table S20). In addition, 74% reported that they frequently view the images of the radiology test requested (Additional file 1: Table S21). However, 26% reported that they were not confident in communicating the results to the patient (Additional file 1: Table S22). When asked what they would do if their shift ended, and they had requested a radiology exam which was expected to be performed within 8 h of their shift ending, 51% reported that at the end of the shift they hand over the information to the team that is taking over; 36% reported that would check the following day, 7% would follow up the exam from home, and 6% would go home without checking (Additional file 1: Table S23).

#### Open comments from the quantitative survey

In the questionnaire, interns and radiologists were asked to highlight any topics not covered in the survey which they considered important for inclusion in a preparatory course (Additional file 1: Appendix B). The following themes emerged:

(A) Required clinical information when requesting studies and patient preparation for radiology procedures.
(B) Working with information technology systems for requesting and viewing radiological examinations and communication with radiologists and radiographers.
(C) Use of contrast material in radiology and radiation protection.
(D) Interpreting studies specifically decoding radiology terminology and how to communicate imaging results to patients.

### Group concept mapping (GCM)

#### GCM strategy and cluster map construction

Sixty-nine participants submitted responses identifying topics for inclusion in a preparatory course for radiology. Following removal of exact duplicate responses, 87 non-duplicate statements were included; 56.5% (*n* = 39) of the respondents during this stage were male, and the remaining 43.5% (*n* = 30) were female; 15.9% (*n* = 11) of respondents were consultant radiologists, 34.7% (*n* = 24) consultant grade doctors in other specialties, 15.9% (*n* = 11) specialist registrars (across a variety of specialties), 14.5% (*n* = 10) senior house officers, 4.3% (*n* = 3) registrars, and the remaining respondents (14.5%, *n* = 10) included medical educators, interns, and unspecified.

**Fig. 1:**
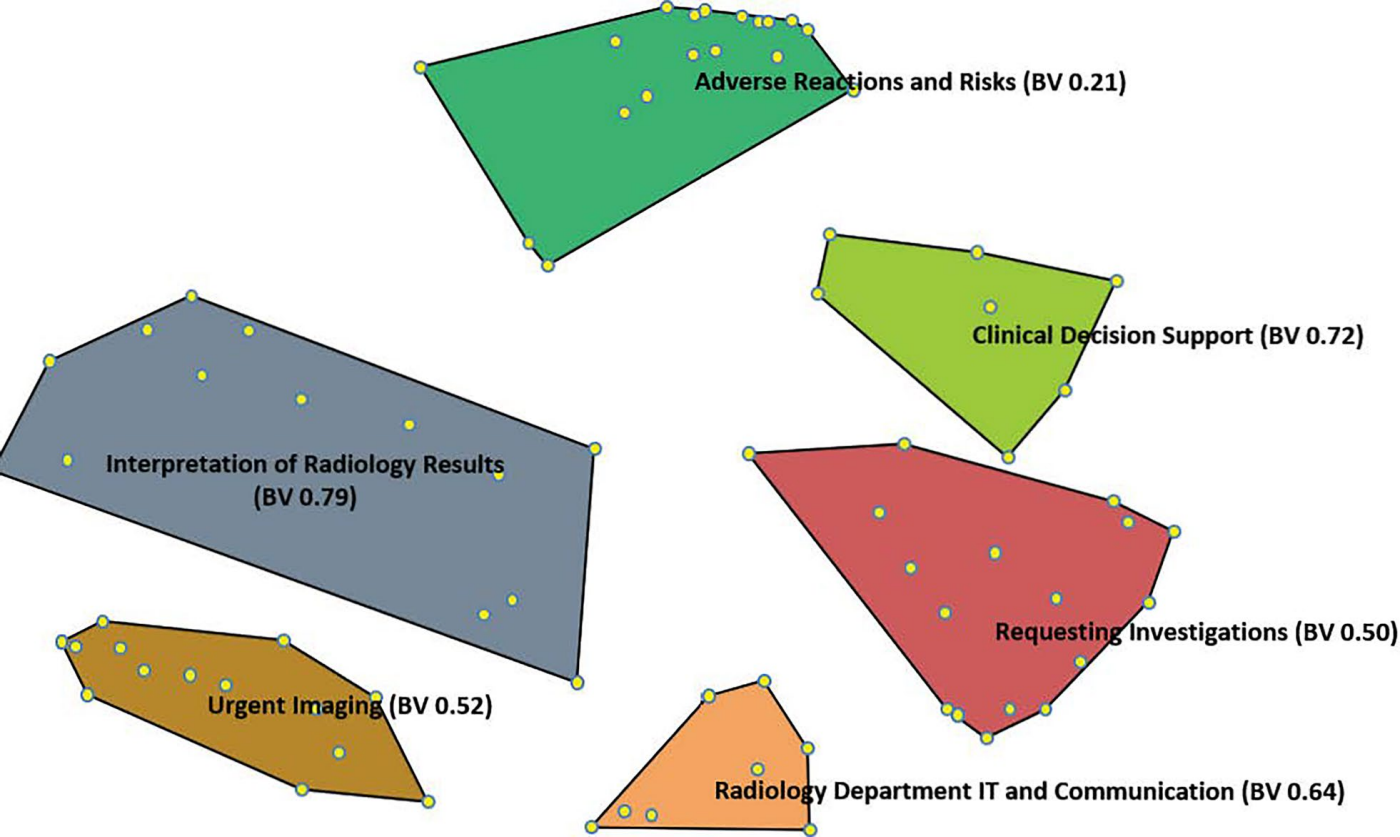
Cluster map of key learning objectives for a preparatory course. Multidimensional scaling and hierarchical cluster analysis revealed six thematic clusters. Each individual point corresponds to a statement (*n* = 87). The closer statements are to each other, the closer in meaning they were perceived to be by participants performing the sorting. Bridging values (BV) for each cluster are shown in parentheses (see Additional file 1: Appendix C)

Following analysis of the statement sortings, the following six thematic clusters were generated (Fig. 1, Additional file 1: Appendix C).

- Cluster 1: Requesting Investigations—how to request imaging examinations with emphasis on relevant clinical information.
- Cluster 2: Clinical Decision Support—understanding appropriate imaging modalities, which are necessary and add value in diagnosis and patient management.
- Cluster 3: Radiology Department IT and Communication—communicating with radiologists and radiographers to justify the requested imaging examination.
- Cluster 4: Adverse Reactions and Risks—contrast media and radiation.
- Cluster 5: Interpretation of Radiology Results.
- Cluster 6: Urgent imaging—Prioritising examinations into urgent and non-urgent categories based on clinical grounds. Understanding how the radiology department prioritises examinations.

## Discussion

Consistent with previous studies, our national survey demonstrated that undergraduate medical training does not adequately prepare newly qualified doctors for their internship year [3, 12]. This once again highlights the urgent need for novel approaches in the design of preparation for practice courses. Although GCM does not validate the national survey findings it supported the findings and created six thematic clusters which may serve as valuable signposts when designing a radiology preparatory module. For each thematic cluster, a body of existing evidence has studied the importance of that domain in the provision of radiological services which is discussed forthwith.

Appropriate requesting and use of imaging studies is a vital skill that should form an integral part of medical education [16]. In this study, most interns reported that they were frequently uncertain regarding the indication for radiology examinations when completing a request form with many encountering difficulties in deciding which study to choose. This concern was also identified in the open comments from the quantitative survey. This is consistent with other studies where a lack of understanding regarding the appropriate use of the various imaging modalities has been reported [17]. Concordantly, the need to address this deficiency was identified in the GCM with a sample statement being *The importance of completing requests for radiology properly including what question needs to be answered. Selecting the correct investigation to solve the clinical problem* (Additional file 1: Appendix C). On the point cluster map, Clinical Decision Support is in immediate proximity to the Requesting Investigations cluster indicating that there are thematically similar and should be addressed together in a preparatory course. Clinical decision support refers to helping referrers understand which imaging modalities are most appropriate in the diagnosis and management of individual patients. Therefore, these clusters are complementary.

The inclusion of the Adverse Reactions and Risk cluster which specifically addresses the use of contrast media and radiation protection was supported by the fact in the national survey, only 52% interns rated their understanding of the use of contrast media to be adequate. In addition, most radiologists, 86%, reported that they felt that interns did not understand the appropriate use of contrast media for radiology investigations. This uncertainty as to which studies require contrast media, when to use premedication and the risks associated with contrast has been previously described [18]. Moreover, for radiation protection, a clear knowledge deficiency was identified with 77% of interns not being familiar with the 10-day rule when imaging patients of childbearing age. Numerous studies have reported that knowledge of radiation related matters among students, interns and non-consultant doctors is suboptimal [19–22]. Radiation is an important component of radiology and should be included in any radiology preparatory module to increase awareness of the associated risks.

Radiology Department IT and Communication was a cluster that overlapped with deficiencies from the survey and was a consistently identified theme that emerged from the open comments. Many interns reported difficulty in preparing patients for imaging tests or interventional procedures and encountered difficulty with the online IT system. In addition, most interns perceived ‘getting the study done’, and ‘communication with the radiologist or radiographer’ as the most difficult challenges they faced when dealing with the radiology department. The UK GMC in their seminal ‘Tomorrow’s Doctors’ publication assert that graduates must be able to communicate in various clinical scenarios, with different departments and must have opportunities to practice communication. A specific strategy to overcome these barriers may include linking simulation and role-playing methods with clinical radiology scenarios [23]. Simulation has been applied with success in teaching interventional procedures, ultrasound, interpretative and non-interpretative skills like communication, management of adverse reactions to contrast media, and in teaching online radiology examination requesting [24–26].

An important finding from the national survey was the discordance between interns and radiologists in their perception of the approachability of the radiology department with a significant percentage of interns viewing the radiology department as unapproachable. This perception may explain why only 1% of interns reported asking the radiologist if they needed information regarding the choice of imaging modality. In a UK study, radiology was mentioned as one of the specialties in which rudeness, dismissive and aggressive behaviours were reported [27]. Part of this ‘unapproachable’ perception may be explained by the frustrations of both radiologists and interns arising from the knowledge deficit of interns. However, it is important to consider the possibility of bullying. Harassment can be devastating for the individual leading to psychological stress, anxiety and depression, thus adversely affecting patient care and safety [28]. When included in a preparatory module, this learning outcome, ‘Radiology Department IT and Communication’, should educate interns on the recognition of workplace bullying and what steps to be taken should it occur. A fundamental question that we have not directly addressed in this study is why are so many of the interactions between referring clinical teams and radiology departments so asymmetric, i.e. between the least-experienced practitioners on one side (interns) and the most-experienced on the other side (consultants)? This asymmetry may contribute to the frustrations experienced by both parties. Therefore, perhaps in addition to preparing students to interact with radiology departments, we should also educate clinical referring teams to respect the relative positions and experience of all involved in these interactions, and ensure that interactions occur at an equivalent, appropriate level.

Within the Urgent Imaging cluster, common areas identified that need to be targeted include *‘How to make an urgent request—who to approach and how’* and ‘*which senior colleagues to consult if there are difficulties/delays in performing the investigation*’. These findings emphasise the importance of structured methods to educate newly qualified doctors about the radiology referral process.

A sample statement from the Interpretation of Radiology Results cluster highlighted the ‘*Importance of seeking results of investigations you have requested*’. Supporting this, only 51% of interns indicated that they would hand over to a colleague information on a requested radiology exam which they expected would be performed within 8 h of their shift ending. Findings suggest that getting the examination performed rather than acting on the results is a priority among interns. This learning outcome may be addressed by educating interns about the importance of the handover related to radiology, highlighting that clinical information which is lost or not communicated promptly may result in compromised patient care and delayed or missed diagnoses [29].

Limitations of this study include a small sample size with a 14% study response rate which may not be truly representative of the target population. Further studies with a larger sample size/higher percentage of participation, could confirm agreement with the study results validating the benefits of this research strategy. A potential shortcoming was that evaluation of interns’ readiness for clinical practice was assessed using subjective self-reported perceptions. In addition, the radiologist respondents may not reflect the point of view of radiology residency programs directors, which should be addressed in future studies. An unexplored area in this study were the differences between medical schools with respect to their educational models for the delivery of undergraduate radiology teaching. Further studies could compare the efficacy of the different educational models in achieving these learning objectives. Of importance, this study doesn’t consider other important radiological learning objectives such as knowledge of the indications, advantages and limitations of various therapeutic interventional radiology procedures; the role of novel digital imaging tools such as artificial intelligence in modern medicine and knowledge of current imaging biomarkers in the management of disease such as CT perfusion in acute stroke. The strength of the study is that few studies specifically address preparation for practice considerations in relation to radiology, and this is the only study that explored the views of both interns and radiologists. Moreover, the ability to examine in parallel the quantitative survey and GCM results help conceptualise the perceived knowledge gaps that need to be addressed to prepare interns for radiology related interactions. Given the centrality of radiology within modern medicine, the thematic clusters identified for a radiology preparatory course may also be applicable to a general preparatory course for newly qualified doctors.

In conclusion, although most interns and radiologists concur that undergraduate radiology teaching is adequate, there is consensus that the knowledge imparted does not prepare interns for interacting effectively with the radiology department following graduation. Deficient areas contributing to this discordance as identified in the national survey, showed significant overlap with the GCM thematic clusters. It is envisaged therefore, that by addressing these six key learning outcomes in a preparation for practice module in radiology, the divide between medical school and clinical practice may be successfully bridged.

## Supplementary Information

**Additional file 1. Supplementary Methods**. The background to Group Concept Mapping, the GCM study setting, participants, process and data analysis are described.** Supplementary Data Appendix A**. Multiple tables showing the quantitative national survey results as follows:** Supplementary Table 1 (S1)**. Adequacy of undergraduate radiology teaching – Interns response.** Supplementary Table 2 (S2)**. Radiology knowledge as compared with other clinical subjects in the undergraduate curriculum.** Supplementary Table 3 (S3)**. Adequacy of undergraduate radiology teaching – Radiologists response.** Supplementary Table 4 (S4)**. Confidence level in the understanding of different imaging modalities and their indication in radiology – Interns response.** Supplementary Table 5 (S5)**. Preparedness of undergraduate medical training for interacting with the radiology department during intern year – Interns response.** Supplementary Table 6 (S6)**. Preparedness of undergraduate medical training for interacting with the radiology department during intern year – Radiologists response.** Supplementary Table 7 (S7)**. Frequency of uncertainty regarding radiology exam indication when completing the request form – Interns response.** Supplementary Table 8 (S8)**. Frequency of uncertainty regarding radiology exam indication when completing the request form – Radiologists response.** Supplementary Table 9 (S9)**. Level of confidence in the indications for radiological studies – Intern response.** Supplementary Table 10 (S10)**. Level of confidence in the indications for radiological studies – Radiologists response.** Supplementary Table 11 (S11)**. Familiarity with 10-day rule in imaging patients of childbearing age.** Supplementary Table 12 (S12)**. Interns understanding of radiation protection – Radiologists response.** Supplementary Table 13 (S13)**. Level of understanding of the use of contrast media for radiology investigations – Intern response.** Supplementary Table 14 (S14)**. Interns understanding of the use of contrast media for radiology investigations – Radiologist response.** Supplementary Table 15 (S15)**. Challenges in dealing with radiology department.** Supplementary Table 16 (S16)**. Approachability of the radiology department – Interns response.** Supplementary Table 17 (S17)**. Approachability of the radiology department – Radiologists response.** Supplementary Table 18 (S18)**. Source of guidance regarding the choice of imaging modality.** Supplementary Table 19 (S19)**. Internet sources for guidance on choice of imaging modality.** Supplementary Table 20 (S20)**. Frequency of checking the result of the requested study – Intern response.** Supplementary Table 21 (S21)**. Frequency of viewing the images of the requested study.** Supplementary Table 22 (S22)**. Level of confidence in communicating radiology results to a patient.** Supplementary Table 23 (S23)**. What do you do if your shift ends, and you have requested a radiology exam which you expect will be performed within 8 hours of your shift ending?** Supplementary Data Appendix B**. Intern and radiologist responses from the open comments section of the national survey.** Supplementary Data Appendix C**. Bridging Values (BV), Importance (I) and Ease of Implementation (E) Ratings for Individual Statements in Each Thematic Cluster.

## Data Availability

All data generated or analysed during this study are included in this published article and its supplementary information files.

## Abbreviations

ANOVA: Analysis of variance
BV: Bridging value
CT: Computed tomography
GCM: Group concept mapping
GMC: General Medical Council
HCA: Hierarchical cluster analysis
IT: Information technology
MDS: Multidimensional scaling
MRI: Magnetic Resonance Imaging
NCHDs: Non-consultant hospital doctors
PHPQ: Preparation for Practice Questionnaire
SD: Standard deviation
