# Supplementary material for: Bridging the divide between medical school and clinical practice: identification of six key learning outcomes for an undergraduate preparatory course in radiology
Source: Insights Imaging. 2021 Feb 12;12:17. doi: 10.1186/s13244-021-00971-1 (PMC7881064; doi:10.1186/s13244-021-00971-1)
Supplement: Supplementary file 1 — Additional file 1. Supplementary Methods. The background to Group Concept Mapping, the GCM study setting, participants, process and data analysis are described. Supplementary Data Appendix A. Multiple tables showing the quantitative national survey results as follows: Supplementary Table 1 (S1). Adequacy of undergraduate radiology teaching – Interns response. Supplementary Table 2 (S2). Radiology knowledge as compared with other clinical subjects in the undergraduate curriculum. Supplementary Table 3 (S3). Adequacy of undergraduate radiology teaching – Radiologists response. Supplementary Table 4 (S4). Confidence level in the understanding of different imaging modalities and their indication in radiology – Interns response. Supplementary Table 5 (S5). Preparedness of undergraduate medical training for interacting with the radiology department during intern year – Interns response. Supplementary Table 6 (S6). Preparedness of undergraduate medical training for interacting with the radiology department during intern year – Radiologists response. Supplementary Table 7 (S7). Frequency of uncertainty regarding radiology exam indication when completing the request form – Interns response. Supplementary Table 8 (S8). Frequency of uncertainty regarding radiology exam indication when completing the request form – Radiologists response. Supplementary Table 9 (S9). Level of confidence in the indications for radiological studies – Intern response. Supplementary Table 10 (S10). Level of confidence in the indications for radiological studies – Radiologists response. Supplementary Table 11 (S11). Familiarity with 10-day rule in imaging patients of childbearing age. Supplementary Table 12 (S12). Interns understanding of radiation protection – Radiologists response. Supplementary Table 13 (S13). Level of understanding of the use of contrast media for radiology investigations – Intern response. Supplementary Table 14 (S14). Interns understanding of the use of contrast media for radiol [file 13244_2021_971_MOESM1_ESM.docx]

**ELECTRONIC SUPPLEMENTARY MATERIAL**

**Supplementary Methods**

**Group Concept Mapping (GCM)**

**Background**

In contrast to other consensus driven research methodologies such as the Delphi method and focus groups, GCM is not based on a prearranged categorization system [13]. Classification and organisation of data is determined by the participants themselves as they work independently of each other. Congruity arises naturally from the data and does not rely on inter-coder discussion to generate a consensus.

**Setting and Participants**

Employing a combination of convenience and snowball sampling techniques, interns, non-consultant hospital doctors (NCHDs), and consultants from multiple specialties were invited to participate in the GCM process via email. Convenience sampling involves recruiting study participants that are readily available instead of from the entire study population. Snowball sampling is where study participants recruit other participants from their peer group. Once the study invitation was accepted, participants were redirected to the group concept system software link (http://www.conceptsystemsglobal.com). Participation in the GCM was considered to indicate consent had been gained. Incentives were not offered to encourage participation.

**GCM Process**

The GCM process was completed by participants on The Concept System © software and comprised five stages: (1) brainstorming (2) sorting of strategies into categories (3) rating strategies on values of ‘importance’ and ‘ease of inclusion’ using a five-point Likert scale (1 = not important to 5 = extremely important) (4) data analysis (5) interpretation of results.

**GCM Data Analysis**

Data analysis employed The Concept System © software which utilises multidimensional scaling (MDS) to create a series of interrelated maps organising the data and hierarchical cluster analysis (HCA) of the MDS coordinates. This two-dimensional composition shows the preparatory topic statements and their relationship to each other through points on the map. The closer the points are together in terms of distance on the map, the closer their relationship to each other. The ’20-to-5’ heuristic was applied to generate the optimal number of thematic clusters [13, 15]. Each cluster and statement within that cluster was also assigned a bridging value between 0 and 1 (BV) [15]. For the individual clusters, the closer the BV to zero, the more participants agreed on the content of that cluster. Descriptive and non-parametric statistical analyses were used for analysis of statement ratings.

**Appendix A. Quantitative National Survey Results**

**Table S1**. Adequacy of undergraduate radiology teaching – Interns response

| **Training Adequacy** | **n** | **%** |
| --- | --- | --- |
| Adequate | 29 | 29.0 |
| Inadequate | 12 | 12.0 |
| Somewhat adequate | 29 | 29.0 |
| Somewhat inadequate | 14 | 14.0 |
| Very adequate | 9 | 9.0 |
| Very inadequate | 7 | 7.0 |
| Total | 100 | 100.0 |

**Table S2.** Radiology knowledge as compared with other clinical subjects in the undergraduate curriculum

| **Knowledge level** | **n** | **%** |
| --- | --- | --- |
| Adequate | 20 | 20.0 |
| Inadequate | 21 | 21.0 |
| Somewhat adequate | 26 | 26.0 |
| Somewhat inadequate | 18 | 18.0 |
| Very adequate | 9 | 9.0 |
| Very inadequate | 6 | 6.0 |
| Total | 100 | 100.0 |

**Table S3.** Adequacy of undergraduate radiology teaching – Radiologists response

|  | **n** | **%** | **Cumulative %** |
| --- | --- | --- | --- |
| Adequate | 6 | 12.0 | 12.0 |
| Inadequate | 12 | 24.0 | 36.0 |
| Somewhat adequate | 18 | 36.0 | 72.0 |
| Somewhat inadequate | 4 | 8.0 | 80.0 |
| Very adequate | 8 | 16.0 | 96.0 |
| Very inadequate | 2 | 4.0 | 100.0 |
| Total | 50 | 100.0 |  |

**Table S4.** Confidence level in the understanding of different imaging modalities and their indication in radiology – Interns response

|  | **n** | **%** |
| --- | --- | --- |
| Very confident | 7 | 7.0 |
| Confident | 22 | 22.0 |
| Somewhat confident | 37 | 37.0 |
| Somewhat unconfident | 18 | 18.0 |
| Not confident | 14 | 14.0 |
| Very unconfident | 2 | 2.0 |
| Total | 100 | 100.0 |

**Table S5.** Preparedness of undergraduate medical training for interacting with the radiology department during intern year – Interns response

|  | **n** | **%** |
| --- | --- | --- |
| Very prepared | 2 | 2.0 |
| Prepared | 6 | 6.0 |
| Somewhat prepared | 26 | 26.0 |
| Somewhat unprepared | 18 | 18.0 |
| Unprepared | 37 | 37.0 |
| Very unprepared | 11 | 11.0 |
| Total | 100 | 100.0 |

**Table S6.** Preparedness of undergraduate medical training for interacting with the radiology department during intern year – Radiologists response

|  | **n** | **%** | **Cumulative %** |
| --- | --- | --- | --- |
| Very unprepared | 1 | 2.0 | 2.0 |
| Somewhat prepared | 19 | 38.0 | 40.0 |
| Somewhat unprepared | 9 | 18.0 | 58.0 |
| Unprepared | 15 | 30.0 | 88.0 |
| Very unprepared | 2 | 4.0 | 92.0 |
| Well prepared | 4 | 8.0 | 100.0 |
| Total | 50 | 100.0 |  |

**Table S7**. Frequency of uncertainty regarding radiology exam indication when completing the request form – Interns response

|  | **n** | **%** |
| --- | --- | --- |
| Very frequently | 4 | 4.0 |
| Frequently | 14 | 14.0 |
| Somewhat frequently | 35 | 35.0 |
| Somewhat infrequently | 29 | 29.0 |
| Infrequently | 17 | 17.0 |
| Very infrequently | 1 | 1.0 |
| Total | 100 | 100.0 |

**Table S8**. Frequency of uncertainty regarding radiology exam indication when completing the request form – Radiologists response

|  | **n** | **%** | **Cumulative %** |
| --- | --- | --- | --- |
| Frequently | 19 | 38.0 | 38.0 |
| Not frequently | 2 | 4.0 | 42.0 |
| Somewhat frequently | 15 | 30.0 | 72.0 |
| Somewhat infrequently | 2 | 4.0 | 76.0 |
| Very frequently | 12 | 24.0 | 100.0 |
| Total | 50 | 100.0 |  |

**Table S9**. Level of confidence in the indications for radiological studies – Intern response

|  | **Very confident** | **Confident** | **Somewhat confident** | **Somewhat unconfident** | **Unconfident** | **Very unconfident** |
| --- | --- | --- | --- | --- | --- | --- |
| Plain film | 25 | 49 | 19 | 3 | 2 | 2 |
| Ultrasound | 18 | 30 | 23 | 13 | 9 | 7 |
| CT | 13 | 24 | 41 | 12 | 7 | 3 |
| MRI | 6 | 14 | 42 | 23 | 10 | 5 |
| PET CT | 2 | 14 | 22 | 28 | 21 | 13 |
| Nuclear medicine | 1 | 2 | 17 | 31 | 27 | 22 |

**Table S10**. Level of confidence in the indications for radiological studies – Radiologists response

| **Modalities** | **Very unconfident** | **Not confident** | **Somewhat unconfident** | **Somewhat confident** | **Confident** | **Very confident** |
| --- | --- | --- | --- | --- | --- | --- |
| Plain film | 2 | 5 | 5 | 21 | 14 | 3 |
| CT | 3 | 7 | 19 | 18 | 2 | 1 |
| MRI | 3 | 8 | 18 | 14 | 7 | 0 |
| Ultrasound | 6 | 15 | 19 | 8 | 2 | 0 |
| Nuclear medicine | 10 | 22 | 11 | 5 | 2 | 0 |
| Interventional radiology | 12 | 22 | 11 | 3 | 1 | 0 |

**Table S11.** Familiarity with 10-day rule in imaging patients of childbearing age

|  | **n** | **%** |
| --- | --- | --- |
| Very familiar | 1 | 1.0 |
| Familiar | 8 | 8 |
| Somewhat familiar | 14 | 14.0 |
| Somewhat unfamiliar | 9 | 9.0 |
| Unfamiliar | 37 | 37.0 |
| Very unfamiliar | 31 | 31.0 |
| Total | 100 | 100.0 |

**Table S12.** Interns understanding of radiation protection – Radiologists response

|  | **n** | **%** | **Cumulative %** |
| --- | --- | --- | --- |
| Adequate | 3 | 6.0 | 6.0 |
| Inadequate | 13 | 26.0 | 32.0 |
| Somewhat adequate | 7 | 14.0 | 46.0 |
| Somewhat inadequate | 14 | 28.0 | 74.0 |
| Very adequate | 1 | 2.0 | 76.0 |
| Very inadequate | 12 | 24.0 | 100.0 |
| Total | 50 | 100.0 |  |

**Table S13.** Level of understanding of the use of contrast media for radiology investigations – Intern response

|  | **n** | **%** |
| --- | --- | --- |
| Very Adequate | 2 | 2.0 |
| Adequate | 16 | 16.0 |
| Somewhat Adequate | 35 | 35.0 |
| Somewhat Inadequate | 29 | 29.0 |
| Inadequate | 15 | 15.0 |
| Very inadequate | 3 | 3.0 |
| Total | 100 | 100.0 |

**Table S14.** Interns understanding of the use of contrast media for radiology investigations – Radiologist response

|  | **n** | **%** | **Cumulative %** |
| --- | --- | --- | --- |
|  | 1 | 2.0 | 2.0 |
| Adequate | 1 | 2.0 | 4.0 |
| Inadequate | 23 | 46.0 | 50.0 |
| Somewhat adequate | 5 | 10.0 | 60.0 |
| Somewhat inadequate | 15 | 30.0 | 90.0 |
| Very inadequate | 5 | 10.0 | 100.0 |
| Total | 50 | 100.0 |  |

**Table S15.** Challenges in dealing with radiology department

|  | **1 (not difficult)** | **2** | **3** | **4** | **5 (difficult)** |
| --- | --- | --- | --- | --- | --- |
| Getting a study done | 5 | 15 | 30 | 30 | 20 |
| Communicating with the  radiographer or radiologist | 8 | 11 | 22 | 28 | 31 |
| The online requesting system | 39 | 33 | 17 | 9 | 2 |
| Deciding which study to choose | 13 | 38 | 40 | 8 | 1 |
| Receiving the results of the test | 25 | 37 | 19 | 13 | 6 |
| Acting on the results of the test | 21 | 25 | 25 | 20 | 9 |
| Preparing patients for a test or intervention | 17 | 25 | 32 | 21 | 5 |

**Table S16.** Approachability of the radiology department – Interns response

|  | **n** | **%** |
| --- | --- | --- |
| Very approachable | 9 | 9.0 |
| Approachable | 19 | 19.0 |
| Somewhat approachable | 24 | 24.0 |
| Somewhat unapproachable | 25 | 25.0 |
| Unapproachable | 16 | 16.0 |
| Very unapproachable | 7 | 7.0 |
| Total | 100 | 100.0 |

**Table S17.** Approachability of the radiology department – Radiologists response

|  | **n** | **%** | **Cumulative %** |
| --- | --- | --- | --- |
| Approachable | 24 | 48.0 | 48.0 |
| Somewhat approachable | 6 | 12.0 | 60.0 |
| Somewhat unapproachable | 6 | 12.0 | 72.0 |
| Very approachable | 12 | 24.0 | 96.0 |
| Very unapproachable | 2 | 4.0 | 100.0 |
| Total | 50 | 100.0 |  |

**Table S18.** Source of guidance regarding the choice of imaging modality

|  | **n** | **%** |
| --- | --- | --- |
| Ask a colleague on your team | 74 | 74.0 |
| Ask a radiologist | 3 | 3.0 |
| Ask your consultant | 5 | 5.0 |
| Look it up on the internet | 11 | 11.0 |
| Other (please specify) | 7 | 7.0 |
| Total | 100 | 100.0 |

**Table S19.** Internet sources for guidance on choice of imaging modality

|  | **n** | **%** |
| --- | --- | --- |
| A specific radiology site such as irefer, ACR, or radiopedia | 32 | 32.0 |
| Other (please specify) | 3 | 3.0 |
| Use a search engine such as google | 46 | 46.0 |
| Total | 100 | 100.0 |

**Table S20.** Frequency of checking the result of the requested study – Intern response

|  | **n** | **%** |
| --- | --- | --- |
| Frequently | 40 | 40.0 |
| Somewhat frequently | 23 | 23.0 |
| Somewhat infrequently | 12 | 12.0 |
| Very frequently | 24 | 24.0 |
| Very infrequently | 1 | 1.0 |
| Total | 100 | 100.0 |

**Table S21.** Frequency of viewing the images of the requested study

|  | **n** | **%** |
| --- | --- | --- |
| Very frequently | 13 | 13.0 |
| Frequently | 39 | 39.0 |
| Somewhat frequently | 32 | 32.0 |
| Somewhat infrequently | 14 | 14.0 |
| Infrequently | 2 | 2.0 |
| Total | 100 | 100.0 |

**Table S22**. Level of confidence in communicating radiology results to a patient

|  | **n** | **%** |
| --- | --- | --- |
| Very confident | 6 | 6.0 |
| Confident | 37 | 37.0 |
| Somewhat confident | 31 | 31.0 |
| Somewhat unconfident | 19 | 19.0 |
| Unconfident | 3 | 3.0 |
| Very unconfident | 4 | 4.0 |
| Total | 100 | 100.0 |

**Table S23.** What do you do if your shift ends, and you have requested a radiology exam which you expect will be performed within 8 hours of your shift ending?

|  | **n** | **%** |
| --- | --- | --- |
| Check the next day at work | 36 | 36.0 |
| Follow up the exam from home | 7 | 7.0 |
| Go home | 6 | 6.0 |
| Hand over the information to the team taking over | 51 | 51.0 |
| Total | 100 | 100.0 |

**Appendix B. If you were asked to design an intern preparatory course for interacting with the radiology department, are there any topics not covered in this survey which you feel are important?**

**Intern Responses**

1. A list of information the radiologist is likely to require would be useful.
2. An induction talk from the in-hospital radiologists.
3. Communicating results to a patient.
4. Dealing with online requesting system.
5. Discussing scans and contrast.
6. Radiology should have a course on how to deal with other healthcare professionals as they are notorious for their unwillingness to cooperate with other teams.
7. How to argue your case with radiologists, they are extremely unhelpful and try to avoid doing CTs at all costs, will redirect you to every other modality of imaging.
8. How to make a case for a scan when discussing with the radiologist.
9. How to not get berated by radiology i.e. Ensure you are well prepared with results and for the rudeness that you may be presented with.
10. How to present clinical questions in a meaningful way to radiologists so that they would be able to help with the patient’s case.
11. How to present relevant details that the radiologist wants to know.
12. Go through the process of writing a report.
13. Indications.
14. Indications for scans and what the radiologist likes to see.
15. Interventional Radiology: indications and patient preparation.
16. When to use contrast and preparing patients for scans.
17. Making a scan request. Further information on contrast versus non-contrast.
18. Radiation protection
19. Run through ISBAR type scenarios for communicating with radiologists.
20. Create a per hospital guide as to how to communicate with radiology, info on when to contact them and how night-time imaging requests work in each hospital e.g. consultant to consultant or Reg to consultant etc.
21. Tips and tricks for reading CTs and common findings to for.
22. Structured approach to vetting scans (what info to present/have on hand).
23. Terminology of scans e.g. hypoattenuation vs hypodensity etc and what they mean.
24. What clinical information radiographers require?
25. What the radiologist what to know.
26. What is required to prepare a patient for different radiological procedures e.g. CTPA, renal biopsy etc.
27. Why do patients need to fast for certain procedures?

**Radiologist Responses**

1. Appropriate clinical information is essential.
2. Awareness of waiting lists and importance of minimizing unnecessary examinations.
3. Being prepared to discuss the case as opposed to 'oh I didn’t see the patient'.
4. Choosing and using radiology investigations.
5. Contrast media
6. Every medical staff need to understand that newer techniques does not mean better techniques. They are simply more specialised to answer specific questions and basic radiological techniques as plain x-ray and US should not be abandoned.
7. Explaining procedures to the patient.
8. Fasting patients pre-ultrasound abdomen, filling bladder pre-TA pelvis. Understanding MRI contraindications. Understanding why multiphase CT may sometimes be required. Sufficient iv access such as exams which require a larger cannula to facilitate faster flow rates.
9. How to ignore instructions from the "team" and think for themselves.
10. How to put the information we need to know on the request form.
11. Patient’s Clinical information.
12. Radiation safety.

**Appendix C. Bridging Values (BV), Importance (I) and Ease of Implementation (E) Ratings for Individual Statements in Each Thematic Cluster**

| Cluster 1: Requesting Investigations | | | BV | I | E |
| --- | --- | --- | --- | --- | --- |
|  | 21. | How to accurately complete a request form i.e. how to select and enter informative clinical data | 0.33 | 4.73 | 4.50 |
|  | 24. | How to fill out a request card | 0.33 | 4.64 | 4.58 |
|  | 44. | Components of a radiology request | 0.33 | 4.36 | 4.75 |
|  | 63. | How to accurately complete a request form | 0.33 | 4.70 | 4.58 |
|  | 57. | Correct completion of content of request form | 0.34 | 4.82 | 4.58 |
|  | 82. | Importance of filling out request form properly | 0.38 | 4.82 | 4.50 |
|  | 35. | The importance of completing requests for radiology properly including what question needs to be answered. Selecting the correct investigation to solve the clinical problem. | 0.39 | 4.64 | 4.42 |
|  | 59. | Requesting tests | 0.40 | 4.64 | 4.55 |
|  | 5. | Explicitly stating why a test is being done i.e. what is the clinical question/hypothesis being 'tested' by performing this radiological procedure | 0.48 | 4.82 | 4.42 |
|  | 8. | Clinical question relevant to the radiologist to allow appropriate test and to aid interpretation | 0.48 | 4.45 | 4.42 |
|  | 1. | Emphasis on which test is most suitable for which clinical scenario | 0.54 | 4.64 | 4.33 |
|  | 40. | Decision support guidance | 0.56 | 3.82 | 4.00 |
|  | 23. | Clarify the purpose of the scan(s), and if the modality and urgency of scan is appropriate for the clinical question. | 0.58 | 4.45 | 4.09 |
|  | 20. | When and with whom to check if the investigation is necessary or likely to change management | 0.59 | 4.45 | 4.00 |
|  | 80. | Linking the patient's clinical picture and their lab investigations with the need for investigations | 0.60 | 4.40 | 4.17 |
|  | 34. | Knowledge about how investigation is done and is the patient fit for it, know what alternative investigations are available | 0.63 | 4.27 | 3.91 |
|  | 7. | Which imaging techniques are most appropriate for aiding diagnosis and who best to inform or decide on that key question | 0.63 | 4.36 | 4.17 |
|  | 60. | Indications for different scans | 0.66 | 4.45 | 4.17 |
|  | 56. | Which imaging techniques are most appropriate for aiding diagnosis | 0.70 | 4.45 | 4.00 |
|  | 15. | The importance of seeking the results of investigations that you have requested | 0.70 | 4.73 | 4.58 |
|  |  | **Count: 20 Mean:** | **0.50** | **4.53** | **4.34** |
|  |  | **Standard Deviation:** | **0.1** | **0.2** | **0.3** |

| Cluster 2: Clinical Decision Support | | | BV | I | E |
| --- | --- | --- | --- | --- | --- |
|  | 32. | Emphasize the importance of completing the clinical information (including relevant comorbidities such as renal function, etc.) | 0.64 | 4.73 | 4.67 |
|  | 74. | How to consent patients | 0.69 | 4.45 | 4.25 |
|  | 3. | Over-testing of patients e.g. CT brain studies done in patients with active epilepsy every time they present to the ED | 0.70 | 4.18 | 3.92 |
|  | 46. | Indications and contraindications of various imaging techniques | 0.73 | 4.45 | 3.75 |
|  | 71. | IV requirements | 0.74 | 4.27 | 4.09 |
|  | 12. | Requesting the correct test in paediatric patients e.g. spinal ultrasound versus MRI dependent on their age | 0.76 | 3.82 | 3.33 |
|  | 36. | Accurate clinical information and demographic information is vital | 0.79 | 4.27 | 4.42 |
|  |  | **Count: 7 Mean:** | **0.72** | **4.31** | **4.06** |
|  |  | **Standard Deviation:** | **0.05** | **0.28** | **0.44** |

| Cluster 3: Radiology Department IT and Communication | | | BV | I | E |
| --- | --- | --- | --- | --- | --- |
|  | 28. | How to discuss a scan. How to discuss the most suitable scan. How to discuss an interventional procedure. | 0.53 | 4.18 | 4.17 |
|  | 67. | Prioritising requests to radiologists | 0.55 | 4.09 | 3.92 |
|  | 49. | Use of relevant IT systems for requesting and retrieving reports | 0.58 | 4.55 | 4.17 |
|  | 29. | Respect the radiology department by providing as much helpful clinical information to aid diagnosis, improve efficiency and write with clear handwriting on request forms. | 0.61 | 4.45 | 4.75 |
|  | 30. | CT COLONOGRAPHY the patient needs to present with the request on the day of request so the patient can collect the prep pack and save a journey | 0.63 | 3.64 | 4.33 |
|  | 43. | How to follow up and act on the results of the examination | 0.72 | 4.64 | 4.42 |
|  | 72. | Understanding the simple X ray imaging, how to escalate the care based on the findings, which are urgent and which situations can await | 0.74 | 4.45 | 3.92 |
|  | 78. | How to interpret a radiology report correctly | 0.77 | 4.45 | 4.00 |
|  |  | **Count: 8 Mean:** | **0.64** | **4.31** | **4.21** |
|  |  | **Standard Deviation:** | **0.09** | **0.33** | **0.28** |

| Cluster 4: Adverse Reactions and Risks | | | BV | I | E |
| --- | --- | --- | --- | --- | --- |
|  | 54. | Managing anaphylactic resections | 0.00 | 4.82 | 4.50 |
|  | 61. | Managing contrast nephropathy and reaction/allergy | 0.00 | 4.55 | 4.33 |
|  | 83. | How to manage anaphylactic resections | 0.00 | 4.73 | 4.50 |
|  | 58. | Patient safety | 0.01 | 4.60 | 4.58 |
|  | 25. | Safe administration of contrast | 0.01 | 4.27 | 4.08 |
|  | 42. | imaging of potentially pregnant patient policy | 0.01 | 4.55 | 4.58 |
|  | 84. | Safe practice when in the diagnostic imaging department | 0.02 | 4.45 | 4.17 |
|  | 53. | Safe practice in use of radioactive materials for diagnostic imaging | 0.03 | 3.64 | 4.08 |
|  | 31. | Risk factors for contrast nephropathy | 0.04 | 4.18 | 4.25 |
|  | 39. | specialised paediatric examinations and radiation dose for children | 0.09 | 4.00 | 3.75 |
|  | 19. | How to assess/triage the risk of CMN in patients with CKD | 0.11 | 4.00 | 3.83 |
|  | 37. | Rad protection. Importance of creatinine and contrast scans. Importance of clinical information, up to date info bloods etc. | 0.11 | 4.40 | 4.58 |
|  | 55. | Safe practice when diagnostic imaging | 0.20 | 4.18 | 3.91 |
|  | 13. | Being aware of radiation risks to paediatric patients | 0.24 | 4.64 | 4.25 |
|  | 41. | importance of renal function to examinations | 0.31 | 4.45 | 4.75 |
|  | 77. | incorporating an understanding of risk benefit associated with radiological investigations | 0.31 | 4.45 | 3.83 |
|  | 11. | Risks, benefits, and alternatives to common radiological investigations and interventions | 0.40 | 3.73 | 4.00 |
|  | 45. | Data protection issues regarding the use of patient scans (e.g. photos taken on smartphones, presentations at educational activities, etc.). | 0.66 | 4.00 | 4.67 |
|  | 33. | Know what bloods are required and what medications are relevant pre radiology intervention, | 0.74 | 4.55 | 4.33 |
|  | 76. | cost to hospital and patient (time involved) of investigation | 0.84 | 3.55 | 3.42 |
|  |  | **Count: 20 Mean:** | **0.21** | **4.29** | **4.22** |
|  |  | **Standard Deviation:** | **0.26** | **0.37** | **0.36** |

| Cluster 5: Interpretation of Radiology Results | | | BV | I | E |
| --- | --- | --- | --- | --- | --- |
|  | 22. | Develop an understanding of how a radiologist triages scans by clinical priority and the many constraints surrounding same - time, staff, cost, radiation | 0.59 | 3.09 | 3.58 |
|  | 14. | How to be competent in high level evaluation for simple investigations | 0.61 | 3.82 | 3.67 |
|  | 10. | Reviewing common X-rays on call (e.g. chest X-ray, ng tube placement, PFA, CT brain) | 0.61 | 4.70 | 4.33 |
|  | 65. | How to discuss the most suitable scan | 0.61 | 4.09 | 4.17 |
|  | 6. | Understand how radiologists triage scans by clinical priority, constraints-time, staff, radiation. | 0.65 | 3.64 | 3.67 |
|  | 70. | Knowing what constitutes an "act now" red flag report from radiology | 0.79 | 4.64 | 4.55 |
|  | 2. | The frequency of incidental findings | 0.84 | 2.64 | 3.50 |
|  | 75. | The need for a collaborative approach to diagnosis | 0.85 | 4.00 | 3.92 |
|  | 73. | Clear pathways which teams should follow based on imaging findings | 0.86 | 4.09 | 3.92 |
|  | 4. | Understanding the limits of certain tests- CT brain, CXR | 0.86 | 4.45 | 3.92 |
|  | 9. | How to interpret CT Brain | 0.93 | 3.91 | 3.75 |
|  | 16. | The importance of entering commentary on conclusions derived from an Xray report in the clinical notes | 0.93 | 4.09 | 4.75 |
|  | 17. | The importance of reviewing films at a structured MDT | 0.99 | 3.82 | 4.33 |
|  | 62. | Importance of seeking results of investigations you have requested | 1.00 | 4.64 | 4.58 |
|  |  | **Count: 14 Mean:** | **0.79** | **3.97** | **4.05** |
|  |  | **Standard Deviation:** | **0.15** | **0.58** | **0.41** |

| Cluster 6: Urgent Imaging | | | BV | I | E |
| --- | --- | --- | --- | --- | --- |
|  | 81. | Knowing occasions when personal contact with the radiologist is necessary e.g. interventional cases | 0.38 | 4.36 | 4.17 |
|  | 79. | How to work with radiologists to arrange best test for patients | 0.39 | 4.70 | 4.17 |
|  | 38. | Need to respect radiographer and radiologist workplace - appreciate that every disturbance can be a hindrance and a distraction | 0.39 | 4.36 | 4.42 |
|  | 50. | Dealing with difficult colleagues | 0.40 | 3.82 | 3.25 |
|  | 52. | Communication skills for making verbal requests | 0.40 | 4.00 | 4.00 |
|  | 69. | Handling consultant to consultant requests (being the mediator) | 0.49 | 3.91 | 4.00 |
|  | 47. | Dealing with radiology out of hours | 0.50 | 4.36 | 4.00 |
|  | 68. | Knowing when to approach the registrar and when consultant review is needed | 0.52 | 4.36 | 3.92 |
|  | 48. | How to make an urgent request - who to approach and how | 0.53 | 4.64 | 4.42 |
|  | 51. | How the radiology department is structured and how it works - where do requests go, who reviews them and prioritises them, the phases of reporting etc. | 0.54 | 3.91 | 4.25 |
|  | 64. | How to discuss a scan | 0.58 | 4.27 | 4.25 |
|  | 27. | Learning interpersonal skills to deal with the personalities you encounter in radiology | 0.59 | 3.73 | 3.18 |
|  | 66. | How to discuss an interventional procedure | 0.69 | 4.00 | 4.00 |
|  | 18. | Knowing which senior colleagues to consult if there are difficulties/delays in performing the investigation | 0.72 | 4.27 | 4.25 |
|  | 26. | Clarification with the primary team (their own) as to what the purpose of the scan is | 0.75 | 4.18 | 4.09 |
|  |  | **Count: 15 Mean:** | **0.52** | **4.19** | **4.02** |
|  |  | **Standard Deviation:** | **0.12** | **0.29** | **0.36** |
